# Promoting youth well-being: a qualitative study of Finnish YouTubers’ mental health content

**DOI:** 10.1093/heapro/daaf074

**Published:** 2025-06-12

**Authors:** Tuula Nygård, Pirjo Lindfors

**Affiliations:** Unit of Health Sciences, Faculty of Social Sciences, Tampere University, 33014 Tampere, Finland; Information Studies, Faculty of Humanities, University of Oulu, 90014 Oulu, Finland; Unit of Health Sciences, Faculty of Social Sciences, Tampere University, 33014 Tampere, Finland

**Keywords:** mental health, qualitative descriptive design, social media influencer, YouTube, young people

## Abstract

Social media has become an integral part of young people’s daily lives. Influencers, such as YouTubers, can serve as role models who shape their followers’ perceptions and management of mental health. This study analyzes the mental health content and characteristics of popular Finnish YouTubers who, except one, were not mental health professionals and were not primarily identified as health influencers. Data were collected from 19 YouTube channels between April 2023 and April 2024. Using qualitative descriptive analysis, we examined 1 987 vlogs, 161 of which contained mental health content. The YouTubers predominantly shaped their personal mental health challenges through recovery narratives. Emotional self-disclosure fosters authenticity, strengthens the parasocial relationship, and positions the YouTubers as both peer supporters and experiential experts. Anxiety and depression were the most frequently discussed mental health topics, along with panic disorder, eating disorder, physical appearance, and self-esteem. They offered numerous tips for supporting mental well-being, highlighting the importance of not being alone, seeking help, and promoting self-compassion and self-care strategies in alignment with positive psychology principles. These findings deepen our understanding of how mental health information can be communicated in ways that resonate with young people, providing them with valuable resources for social support and mental health promotion. Collaborating with YouTubers in health campaigns and interventions can help health promotion organizations reach young people who may be less receptive to traditional programs, ultimately contributing to greater mental health equity.

Contribution to Health Promotion

- By sharing intimate mental health recovery narratives, the YouTubers created personal, emotionally resonant, and relatable content. This positions them as experiential experts and peer supporters within young audiences’ digital communities.
- The findings of this study deepen our understanding of the types of social support young people seek and how mental health information can be adapted to better engage and resonate with them.
- Health promotion organizations may benefit from partnering with YouTubers in mental health campaigns and interventions to reach young people who are typically less responsive to traditional health promotion programs, thereby promoting mental health equity.

## INTRODUCTION

For young people, social media is an inseparable part of everyday life, not simply a digital space apart from the ‘real’ world. Social media offers positive experiences and peer support, facilitates the formation and maintenance of social relationships, and serves as a platform for producing and accessing health and well-being information (Abidin 2015, Naslund *et al.* 2020, Popat and Tarrant 2023), all of which are key contributors to promoting positive mental health. As young people increasingly seek out social media for health-related information (Goodyear *et al.* 2018, O’Reilly *et al.* 2018a), social media influencers (SMIs), such as YouTubers, have emerged as important sources of health content. SMIs, who may have hundreds of thousands or even millions of followers, play a powerful role in shaping young people’s personal and social identities (Subrahmanyam and Michikyan 2022, Wilska *et al.* 2023). They influence how youth perceive and engage with mental health topics during a critical stage of physical, cognitive, and socioemotional development. Influencer culture is particularly impactful among youth: three in four young Americans aged 18–29 follow influencers on social media, compared with 44% in the next age group and ∼20% among older adults (Faverio and Anderson 2022, Adeane and Stasiak 2024).

However, SMIs are often perceived as unreliable sources of health information, largely due to their lack of formal qualifications and their tendency to portray highly curated, commercialized lifestyles (Hendry *et al.* 2022). To date, research on SMIs and youth health has primarily emphasized negative impacts and associations, often employing quantitative research designs. Meanwhile, the topic of mental health has remained underexplored in this context (Engel *et al.* 2024). Therefore, there is a growing need for qualitative research to examine in greater detail how SMIs engage with mental health topics and communicate about mental health challenges.

### Mental health promotion, YouTube, and SMIs

According to the World Health Organization (WHO 2004), mental health promotion refers to the process of enabling people to gain greater control over and improve their mental health. This includes creating supportive living conditions and environments, empowering individuals and communities to make choices that promote mental health, and strengthening internal and external resources such as resilience and social support. Both the internet’s role as a source of mental health support (Lupton 2021) and the use of experiential knowledge, such as peer support, in mental health promotion are now well-established practices (Dennis 2003, Barton and Henderson 2016). Furthermore, the principles of positive psychology (Seligman and Csikszentmihalyi 2000, Barry 2009) are closely linked to mental health promotion, as both emphasize enhancing well-being and personal strengths. Positive mental health includes not only positive emotions but also personal resources such as self-esteem, optimism, a sense of mastery, and the ability to cope with adversity (Barry 2009). It also includes dimensions of social and peer support, which are built on lived experience and experiential knowledge (Evans 2023). Exploring these dimensions through the lens of YouTubers promoting mental health is especially relevant. Among social media platforms, YouTube dominates the online landscape. However, platforms such as TikTok, Instagram, and Snapchat are increasingly popular among young people (Auxier and Anderson 2021). Globally, YouTube has 2.7 billion monthly users, with individuals aged 15–35 representing the largest demographic (Global Media Insight 2024). In Finland, ∼90% of 12–18-year-olds use YouTube regularly (Ebrand Group 2022). While shorter videos are now available, YouTube’s core strength remains its long-form content, which allows for in-depth and versatile exploration of topics like mental health.

Young people primarily use digital media for information, entertainment, peer interaction, and communication (Subrahmanyam and Greenfield 2008, Valkenburg and Peter 2011, Anderson and Jiang 2018). They turn to YouTube for various health-related purposes, such as seeking answers to health questions, exploring available services, and finding social support (Mohamed and Shoufan 2024). Notably, young users are often exposed to health-related content passively even when they are not actively seeking it (Harris *et al.* 2021). Given that many young people may be uncomfortable sharing personal health issues online, anonymity becomes a vital feature of social media, allowing for more accessible engagement. For example, teenage boys may explore mental health topics on social media that they feel unable to discuss elsewhere (Best *et al.* 2014). Public health organizations and nongovernmental organizations (NGOs) have created numerous YouTube channels that offer credible, anonymous, and easily accessible mental health information (Mohamed and Shoufan 2024). As such, YouTube plays a significant role in shaping users’ understanding of health, influencing both behaviors and decision-making processes (Haslam *et al.* 2019).

SMIs, such as YouTubers, are typically everyday individuals who successfully brand themselves and their channels, often as lifestyle, beauty, or gaming vloggers. They often share personal and intimate aspects of their lives, attracting substantial followings and visibility (Abidin 2015). SMIs can become perceived as health influencers even if they do not explicitly brand themselves as such (Hendry *et al.* 2022). Unlike licensed health professionals, SMIs are not subject to formal regulations. Instead, their perceived credibility is validated by audience engagement, through likes, shares, comments, and other interactive metrics (Hendry *et al.* 2022, Wickström and Lind 2024). Celebrity influencers often collaborate with advertising agencies and brands. In such partnerships, SMIs’ role as opinion leaders is utilized in corporate marketing campaigns (Ember 2015, Lee and Watkins 2016, Reinikainen *et al.* 2020), and they are expected to demonstrate expertise as part of their brand identity (Hendry *et al.* 2022). To enhance commercial success, influencers strive to increase follower engagement by posting regularly and encouraging interaction with their content (Farivar *et al.* 2022).

### Parasocial interaction, trust, and authenticity

SMIs expand the reach and engagement of their mental health content by sharing personal experiences with symptoms, treatments, and coping strategies (Engel *et al.* 2024). By presenting mental health narratives that are trustworthy, authentic, emotionally compelling, and framed positively, SMIs can influence their young followers’ attitudes, values, and behaviors (Pfeiffer *et al.* 2014, Jenkins *et al.* 2020, Harris *et al.* 2021, Walker *et al.* 2021, Tuominen 2023). Disclosing personal and intimate stories about mental health struggles can be particularly attractive to followers. These narratives foster trust, convey authenticity, and create a sense of interconnectedness (Wickström and Lind 2024). ‘Real talk’ about mental health has been shown to foster feelings of belonging and acceptance, and enhance perceptions of authenticity among social media users (Abidin 2018, Reade 2021). This sense of authenticity is critical for establishing parasocial relationships. SMIs can meet needs that may go unmet in the ‘real-world’ by sharing health-related content that resonates with their audience’s experiences. This allows influencers to support individuals facing mental health challenges (Marcus *et al.* 2012, Harris *et al.* 2021, Zorell 2022). Moreover, personal narratives of experiences of mental health struggles can help reduce the stigma surrounding these issues (Evans-Lacko *et al.* 2014).

Building on the concept of parasocial relationships, YouTubers often present themselves as virtual friends, siblings, or even loved ones to their followers (Reinikainen *et al.* 2020). While these connections can be perceived as intimate, they remain one-sided and false (Horton and Wohl 1956, Abidin 2015). Nonetheless, followers view these interactions as platforms for social support. This support manifests through peer exchange, sharing coping strategies for daily challenges, and learning from recovery stories (Naslund *et al.* 2014). Therefore, the theory of parasocial relationships can be expanded into the concept of a trans-parasocial relationship, characterized by reciprocal, asynchronous, interactive, and co-created elements of the relationship between influencers and followers (Lou 2022).

SMIs employ interactive strategies, such as addressing the audience directly (by looking into the camera), using the pronoun ‘you,’ and soliciting followers’ feedback, to strengthen their (trans-)parasocial interactions (Horton and Wohl 1956, Hartmann and Goldhoorn 2011, Kim and Song 2016, Penttinen *et al.* 2022, Tuominen 2023). However, according to the parasocial compensation hypothesis, there is a risk that socially isolated individuals may use parasocial relationships to compensate for the lack of real-life connections. This could increase the risk of YouTube addiction (de Bérail *et al.* 2019).

### SMIs’ role in young people’s mental health

Research on SMIs’ roles in young people’s health, especially mental health, remains limited (Engel *et al.* 2024), despite growing interest in public health and health promotion. In their recent review of 51 articles, Engel *et al.* (2024) concluded that most studies have focused on the negative aspects of SMIs’ influence. Only a few studies have explored their potential positive impacts, such as building trustworthy relationships with followers or contributing effectively to health promotion campaigns. Most reviewed studies employed quantitative methods, highlighting a gap in research using qualitative designs. In addition, topics such as mental health and sexual health were underrepresented as were issues relevant to the Global South, marginalized populations, and male audiences.

Research specifically examining how YouTubers discuss mental health remains scarce and often involves small datasets. For instance, in a study of four female Swedish YouTubers, Lind and Wickström (2024) found that video content mainly comprised personal stories of descriptive accounts of mental health challenges. These narratives portrayed mental health issues as manageable, emphasizing personal responsibility and incorporating elements of positive psychology, such as advice on self-improvement, self-care, and seeking help. The authors noted that mental health representations were multilayered and complex and that young audiences interpreted these narratives through the lens of prevailing mental health discourses.

Research investigating how young people (aged 11–18 years) perceive social media concerning mental health suggests that it is often viewed as a risk. Adolescents view social media as potentially harmful to mental well-being, citing concerns such as mood and anxiety disorders, cyberbullying, addiction (O’Reilly *et al.* 2018b), and issues related to validation-seeking, fear of judgment, and body comparison (Popat and Tarrant 2023). Despite their awareness of these risks, many young people struggle to clearly define what mental health means (O’Reilly *et al.* 2018b). Research on followers’ perceptions of the negative effects of YouTubers’ mental health content (Harris *et al.* 2021) has shown that creators often generalize their personal experiences. This generalization may obscure the diversity of mental health challenges faced by viewers, leading to inaccurate self-diagnoses and potentially harmful outcomes, such as overtreatment of conditions like anxiety and depression (Harris *et al.* 2021).

While existing research provides valuable insights into the effects, both positive and negative of SMIs on young people’s mental health, it rarely differs across social media platforms, despite significant variations in content types, formats, algorithms, and target audiences. In addition, studies often overlook how age-related differences in socioemotional and cognitive developments might influence young people’s ability to critically assess social media content.

There is a clear need for more detailed qualitative research on how SMIs frame and discuss mental health and coping strategies with their predominantly young audiences. Given the rising global incidence of youth mental health issues, particularly anxiety and depression (CDC 2022, Kiviruusu *et al.* 2024), this area of reach is especially urgent. It holds potential value for health promotion practitioners and service providers seeking effective ways to engage young people in mental health conversations. The current study addresses this gap by applying a qualitative descriptive approach (Sandelowski 2000, 2010) to analyze vlogs produced by the most popular Finnish YouTubers, as identified by young people with mental health challenges who follow them. To the best of our knowledge, this is the first large-scale study of its kind focused on Finnish YouTubers’ mental health content. A descriptive qualitative analysis is particularly suited for investigating large datasets in under-research areas, allowing for the identification of overarching variation and patterns in the characteristics and content of the data.

This study addresses the following research questions (RQs): (i) What are the characteristics of vlogs that provide mental health content? and (ii) What kinds of mental health content do the most popular Finnish YouTubers create, and how do they discuss mental health?

## MATERIALS AND METHODS

### Setting and participants

This study is part of a larger research consortium examining how young people interpret and use mental health vlogs by popular YouTubers as potential peer support in Finland. YouTuber selection was based on a survey of 255 young people aged 15–29 years, who were recruited through three NGOs focused on youth mental health. Participants were asked to identify which YouTubers they followed and whether they discussed mental health (Pekurinen and Joronen in preparation). We selected the 20 most popular YouTubers identified in this survey. The age range was chosen based on the Finnish Youth Act (2017), which defines individuals younger than 29 years as young people. Furthermore, the ethical guidelines do not require parental consent for participants older than 15 years.

The Ethics Committee of the Tampere Region approved this study on 7 March 2023 (Tampere University n.d.). After data collection and presentation of preliminary results, we informed the YouTubers about the ongoing research. This timing was purposeful, as it allowed us to gather authentic data, given that prior knowledge of the study could have influenced the mental health content produced by the YouTubers. We sent the YouTubers an email containing study information, privacy notices, and the option to opt out of allowing their vlogs to be included in the study. One YouTuber declined participation, resulting in a final sample of 19 participants.

From April 2023 to April 2024, we collected data from 10 female YouTubers, 7 male YouTubers, and 2 couples (each pair considered as single participants). One channel belonged to a minority group (LGBTIQ+). Except for one, none of the YouTubers were trained health professionals. Most YouTubers have been creating content for more than 10 years (Table 1), with follower counts ranging from under 100 000 to almost 1 000 000. All participants were young adults over 18 years of age. Fourteen of the YouTubers were macro-influencers, and five were micro-influencers (Campbell and Farrell 2020), each with commercial partnerships with companies and NGOs. To maintain anonymity, we did not report the exact follower counts or specific content of their vlogs.

**Table 1.** A summary of the YouTubers included in the study.

YouTuber	Vlogging years	Transcripts taken	Type of the vlogs
M1	12	6	Game videos, humor, challenges
M2	13	8	Game videos, humor, challenges
M3	11	9	Game videos, humor, challenges
M4	12	4	Lifestyle, humor, challenges,
M5	17	0	Lifestyle
M6	12	8	Lifestyle, challenges, podcasts
M7	10	21	Facts, news
F1	13	21	Lifestyle
F2	13	1	Lifestyle
F3	12	6	Lifestyle
F4	17	6	Lifestyle
F5	14	0	Lifestyle
F6	6	8	ASMR
F7	11	17	Lifestyle, game videos, GRWM, Q&A
F8	13	2	Everyday life with children
F9	17	23	Lifestyle, GRWM
F10	3	10	Lifestyle
C1	13	0	Everyday life with children, Q&A
C2	9	11	Everyday life with children

We selected vlogs published between early 2020 and April 2024 for analysis to examine content from before and after the COVID-19 pandemic. The authors and a research assistant analyzed 1 987 vlogs, ranging from a few minutes to nearly 2 h in length. We watched all videos in sequence, either in their entirety or fast-forward (e.g. game videos or streaming), and we took notes of their contents, publication dates, views, likes, and comments. Of these, 161 vlogs contained mental health-related content (Table 1). We transcribed the videos using the Microsoft Teams transcription tool, which facilitated the processing of the rich data. For linguistic accuracy, we reviewed all transcripts and transcribed verbatim. Our analysis focused on the mental health contents and key characteristics of the vlogs for all the YouTubers included in the study.

### Data analysis

The qualitative descriptive approach offers a comprehensive summary of the event or phenomenon under study in straightforward, factual terms, making it accessible for understanding among health researchers from various disciplines (Sandelowski 2000, Colorafi and Evans 2016). This approach is well-suited for examining mental health content in vlogs as it allows for clear descriptions of YouTubers’ personal experiences and perceptions (Sandelowski 2010, Sheng *et al.* 2023). In qualitative descriptive studies, qualitative content analysis is an appropriate dynamic analysis strategy because it effectively summarizes the informational content of both verbal and visual data (Sandelowski 2000). It aims to describe the meanings within data, guided by the RQs (Schreier 2012).

We exported the transcribed data to the qualitative data analysis software Atlas.ti. The first author created a preliminary code list, consisting of 30 categories based on observations, notes, and joint discussions (Schreier 2012) (see Supplementary File 1). We then independently coded various data sets using an inductive, data-driven method to identify and label mental health-related content (Luo *et al.* 2022). This approach allowed us to highlight relevant passages, ranging from single phrases to several sentences (Gibbs 2018).

As the analysis progressed, our code list expanded. For instance, we added emotions related to bullying and reasons for bullying under the bullying code. To follow a constructivist approach and encourage a reflexive, consensus-building process, we met weekly to discuss and compare our coding consistency (McKinley and Rose 2019). In addition, we held data sessions where we first coded the data independently and then discussed any differences in interpretation. As the codes were refined, we revisited and recoded previously coded transcripts to incorporate the new codes (Jain and Ogden 1999, Thomas 2006). In total, 158 codes were generated.

After coding, we merged our projects in Atlas.ti, grouped the codes, and identified 15 higher-level categories (see Supplementary File 2). The main data for this study were contained within the subcategories ‘factors contributing to mental well-being’ and ‘factors burdening mental well-being.’ We present the results with translated data extracts, which were slightly edited for readability. Filler words (e.g. ‘like,’ ‘such’) and tautological expressions were removed, unless integral to the context. Our analysis primarily focused on the explicit content of the language, that is, what the YouTubers *said* and the *mental health-related content* they published.

The first author also conducted a descriptive analysis of the vlog thumbnails, which are clickable images displayed on each channel’s homepage. These thumbnails serve as eye-catching preview images that encourage viewers to watch the video. Analyzing the elements (e.g. people, texts, and colors) was part of the broader analysis to understand how the YouTubers presented their mental health-related content and *the contextual characteristics* of those representations.

## RESULTS

### Contextual characteristics of the vlogs

The YouTubers produced various vlogs, including lifestyle (e.g. fitness, travel, fashion), My Week, My Day, Get Ready With Me (GRWM), gaming, challenges, humor, information, Q&A, live streaming, and podcasts (Table 1). The number of views ranged from thousands to millions. Mental health topics were introduced sporadically, not regularly, and varied across channels. Clear distinctions emerged based on the type of YouTuber. Game vloggers, in particular, were less likely to address mental health topics than lifestyle vloggers. While lifestyle vloggers engaged with these topics more frequently, the proportion of such content relative to their overall video output remained low. Notably, none of the YouTubers focused exclusively on mental health; rather, these topics were integrated intermittently within broader content, aligning with each channel’s unique style and profile.

Viewers could not always anticipate that a vlog would include mental health content, as this was not always evident from the title. YouTubers addressed mental health issues in line with the genre of their content, with the exception of gaming videos. Four YouTubers who published gaming videos alongside other content did not address mental health while gaming, with the exception of one YouTuber (M2), who briefly mentioned a mental health discussion forum he had a commercial partnership with.

In lifestyle vlogs, mental health topics such as anxiety and depression were often integrated into other personally experienced content rather than being presented as standalone topics. This approach can help reduce stigma, as it normalizes mental health challenges as part of daily life. For example, two YouTubers discussed mental health while applying makeup in GRWM videos focused on beauty, hairstyling, and fashion routines.

Mental health content was rarely indicated in vlog titles, which were mostly generic (e.g. ‘Comeback’ and ‘All is Well’). Only 38 titles explicitly referenced mental health themes (e.g. ‘My Panic Disorder’ and ‘Depression’), with some addressing personal and difficult topics (e.g. ‘Truly Heavy Losses’ and ‘We Decided to Break Up’). Similarly, the thumbnails did not typically signal mental health issues but instead reflected the YouTubers’ style and the content of the vlogs. The thumbnails generally featured cheerful colors, images, keywords, and illustrations, even in videos addressing mental health. However, some thumbnails were more subdued or in black and white. One thumbnail stood out, featuring a completely black background with the word ‘Addiction’ in white text, marking a clear departure from the YouTuber’s usual style.

### Mental health content of the vlogs

Based on an analysis of the mental health content of the vlogs, three themes emerged: narratives of mental health problems and challenges, promoting mental health in everyday life, and normalizing professional help.

#### Narratives of mental health problems and challenges

The vlogs covered various mental health topics, including anxiety, depression, panic disorder, eating disorder, body image and self-esteem issues, bullying, shame, fears, and stress. Anxiety was the most frequently mentioned, noted by 14 of the 19 participants (Table 2). It was discussed from different perspectives: as general anxiety, a part of another mental health issue, or in the context of specific life events, such as the COVID-19 pandemic and wars. Anxiety was often described as an emotional burden, difficult to define, analyze, or understand as a standalone condition. Instead, it was often framed as a broader sense of discomfort or distress. For example, one participant reflected on their experience: ‘*In high school, I had a lot of anxiety and stress, just feeling bad*’ (M3). This general use of ‘anxiety’ as a vague term for a range of emotional struggles can blur its clinical definition. In the following example, a female YouTuber discusses the possible causes of her anxiety but stops short of identifying a clear cause, instead speculating that stress from her hectic lifestyle and new challenges may be contributing to her anxiety: ‘*It is often difficult to pinpoint exactly what triggers it [anxiety]. But I guess it was just fatigue and stress…It’s been hectic, hectic, with a lot of new things*’ (F1). This tendency to speculate about the causes of anxiety was a recurring theme in the vlogs, reflecting the complexity of identifying its root causes.

**Table 2.** Summary of the coding of the reported themes.

Code	Amount of YouTubers	Amount of vlogs	Entries in Atlas.ti
Anxiety	14	45	99
Depression	8	15	44
Burdensome emotions	15	34	85
Appearance-related problems	10	21	81
Bullying	9	15	48
Health behavior supporting mental health	9	26	63
Pep talk	12	33	54
Recovery stories	6	15	32

Eight YouTubers discussed depression in their vlogs (Table 2). Similar to anxiety, depression was explored from multiple perspectives: as a diagnosed mental disorder related to anxiety, or as a consequence of other mental health issues or life circumstances. One YouTuber shared his experiences with depression in response to a viewer’s post: ‘*I had the same feelings in high school. I was afraid I would be nothing—that I would not find my place in society because I never had a dream job or a clear direction for my life*’ (M3).

The YouTuber positioned himself as a peer supporter, offering reassurance by sharing his own experiences. He linked his depression to the uncertainty of his future and the difficulty of finding a sense of purpose. At the time, the causes of depression appeared more straightforward. In another example, depression was linked to vulnerability in self-esteem and a lack of acceptance from others, particularly in relation to societal norms surrounding body image and physical appearance. One participant captured this connection succinctly: ‘*My depression could really be summed up in one sentence: No one can love a fat girl*’ (F4). Both depression and anxiety were predominantly discussed as past experiences that participants had reflected on, often associating them with various coping strategies.

#### Promoting mental health in everyday life

The YouTubers shared practical advice on coping with mental health challenges and promoting well-being. Their recommendations included mindfulness practices, yoga, meditation, exercise, relaxation techniques, music, quality sleep, and self-care. They began by reflecting on their own journeys of changing their attitudes and behaviors to promote mental well-being. Through self-reflection, they explored their emotions, behaviors, and personal goals. One YouTuber emphasized the importance of letting go of perfectionism, saying: ‘*What if you didn’t always have to strive for perfection? What if less was enough? I am much more than my diploma or my CV. I am good enough just the way I am*’ (F1). Later, F1 discussed learning self-compassion, a process supported by peers who had experienced similar performance pressures. The YouTubers consistently encouraged viewers to adopt positive thinking, practice self-compassion, and embrace unconditionally self-love rather than indulging in negative thoughts or self-blame. F6, for example, emphasized the importance of self-affirmation, stating: ‘*If you have not said today that you’re a great person and that you love yourself, or at least you’re trying hard to love yourself, then say it now. Say it to your body, your experiences, your insecurities. I love myself; I love you, my dear body, and I want to be merciful to myself*’ (F6). Through pep talks, they encouraged viewers to practice self-care, focus on the positives in life, and maintain hope for the future. They conveyed that mental health challenges are temporary and can be overcome. F6 shared an empowering message: ‘*The feeling of anxiety is by no means eternal.’ This moment will pass, I promise you. Even if it feels like all the walls are coming down, it will pass. Trust me*.’

Positive affirmations such as ‘You are valuable,’ ‘Tomorrow will be a better day,’ ‘You are not alone,’ and ‘Everyone is unique and important’ were shared to help buffer difficult emotions and reinforce self-esteem. They also stressed the importance of seeking social support, as isolation can worsen mental health struggles. As M1 explained: *‘It’s not worth staying alone with your thoughts, because it can be harmful. Letting those thoughts and feelings out—talking to close friends or professionals—can really help’* (M1). Thus, the YouTubers also highlighted the invaluable role of family and close friends in providing support.

The YouTubers also reflected on how they changed their behavior to promote better health in response to the problems caused by excessive social media use. These issues were described as overwhelming, with one YouTuber noting: *‘The automated scrolling on social media… fills your brain with useless content, leaving you unable to think clearly because your head is full of distractions*.’ M1 shared how social media addiction negatively affected his mental state, preventing him from enjoying relaxation. As a result, he deleted all games from his computer and only played them when vlogging about them. However, some games, such as Pokémon GO, were described positively as promoting mental well-being, for example, by reducing social isolation.

#### Normalizing professional help

Seeking professional help for various mental health issues was a recurring theme. Therapy, as well as other forms of mental health support, such as services provided by school nurses, occupational health providers, or local health centers, was often recommended as easily accessible and beneficial. YouTubers frequently shared contact information for well-established mental health promotion services, most of which were provided by NGOs. These resources were often included both within the video content and in the video’s information box. Furthermore, therapy and other forms of mental health support were portrayed as vital steps toward personal recovery. One YouTuber shared how therapy helped her break a recurring cycle of emotional distress and how it led to significant life changes. In the following extract, she explains key aspects of therapy, such as processing emotions and understanding the root causes of her distress, without disclosing the specific details of her situation: ‘*My entire life will be completely different because I kind of got over that cycle of feeling bad. I managed to deal with the things that were bothering me, and I understood why I felt bad. Once I dealt with those things, they no longer bother me. I can think about all those things now, and it doesn’t make me feel bad*.’ (F6).

Similarly, a YouTube couple who recovered from depression with the help of antidepressants and therapy emphasized the importance of seeking support, stating: ‘*All depressed people need to seek help because life does not have to be like that. Life can change, but change starts with you*’ (C2).

Some YouTubers expressed that mental health services were essential to their survival. For instance, F4, who had previously struggled with an eating disorder, urged viewers to seek professional help. She emphasized that such illnesses are often insidious and difficult to overcome alone, and stressed that healing begins with the willingness to accept available support. By openly discussing their therapeutic journeys, the YouTubers not only shared the benefits of therapy but also helped reduce the stigma and lowered the threshold for seeking professional help.

At times, YouTubers even recommended therapy as a valuable experience for individuals without a specific diagnosis or crisis, highlighting the general benefits of self-reflection and emotional support. As one YouTuber put it: ‘*I would really recommend it to everyone. If everyone had the opportunity to see a therapist, I would definitely suggest it, even if there isn’t any major problem or crisis, because it’s good to go there for an hour, sit on the couch, and talk about how things are going*’ (F10).

However, despite their overall positive portrayal of mental health services, the YouTubers rarely addressed the real-world challenges of accessing these resources, including within public healthcare systems.

## DISCUSSION

This study is among the first to apply qualitative analysis to large-scale data, examining the characteristics and content of 19 popular YouTuber channels, particularly their mental health-related content. With the exception of one, none of the YouTubers were mental health professionals or identified themselves as health influencers, despite being frequently mentioned by young people who use mental health services. These findings align with Harris *et al.* (2021), who observed that young people often encounter mental health content on social media, even if they are not actively seeking it.

Most of the YouTubers in this study had been vlogging content for several years and had established careers as SMIs, with commercial partnerships across various organizations. However, their mental health content was published sporadically, rather than on a regular schedule, and video thumbnails rarely indicated that mental health topics would be discussed. Our findings suggest that maintaining consistency and integrity in channel profiles is critical for YouTubers, and shapes the content, volume, and frequency of mental health-related posts.

Interestingly, the YouTubers occasionally noted that publishing mental health content felt unusual for them, even though they considered it important. A significant shift in a channel’s profile could jeopardize established relationships with followers, as the profile and personal style are key to building popularity and distinguishing them from other YouTubers (Pöyry *et al.* 2019). From the perspective of commercial collaborators and influencer marketing, engaged followers are seen as both essential and highly valuable (Hendry *et al.* 2022). Thus, YouTubers must carefully curate the mental health content they share, considering both personal context and commercial partnerships involved.

Although the channels and content profiles of the YouTubers varied significantly, ranging from gaming to lifestyle vlogs, their approaches to discussing mental health issues were strikingly similar. Primarily, YouTubers shared their mental health struggles through recovery narratives, portraying these challenges as manageable. This framing aligns with the dominant public discourses on coping and recovery from mental illness (Naslund *et al.* 2020). By sharing intimate, emotionally resonant stories, YouTubers create personal and relatable content that fosters a sense of friendship and reciprocity, enhancing the trans-parasocial experience (Lou 2022). These strategies also enable influencers to position themselves as experiential experts and peer supporters (cf. Dennis 2003), reassuring followers that they are not alone while helping to normalize mental health challenges.

At times, however, YouTubers also described more immediate emotional states, such as feeling low or anxious. These descriptions often included crying and other nonverbal expressions of distress. Consistent with Lind and Wickström’s (2024) observation, this suggests that YouTubers’ posts, despite being edited, reflect their everyday lives and self-disclosure, portraying them as vulnerable and lacking control over their mental health. This authenticity may reinforce perceptions of genuineness. Influencer authenticity, often rooted in personal disclosure (Abidin 2018), is essential for building trust and is highly valued by followers (Adeane and Stasiak 2024). However, it remains a complex construct, especially when the ‘real me’ intersects with YouTubers’ commercial partnerships (Reinikainen *et al.* 2020, Raaper *et al.* 2024). A recent analysis by Adeane and Stasiak (2024) highlights this complexity, showing that while some young people criticize influencers for profiting from mental health-related content, viewing it as morally questionable, others accept sponsored content as an inevitable aspect of YouTubers’ professional practice.

Anxiety and depression emerged as the most common mental health topics in the YouTubers’ content (Table 2), alongside panic disorder, eating disorders, physical image, and self-esteem, all issues likely to resonate with the mental health challenges faced by their young followers. Descriptions of anxiety were often vague and frequently likened to a general sense of feeling unwell. Such comparison, although increasing self-reflection, might help followers understand that feelings of discomfort are a normal part of life and thus manageable and temporary. However, the vlogs provided little detailed explanation of when anxiety might be considered a severe mental health issue requiring professional help. This approach could reflect the concept of ‘concept creep,’ where significant psychological terms are semantically expanded, affecting how they are understood and applied, sometimes as clinical disorders and at other times as broader descriptions of unpleasant states (Haslam *et al.* 2021). While this could help young people recognize symptoms and encourage them to seek help, it may also lead to self-diagnosis or over-diagnosis, potentially putting additional strain on the healthcare system.

When describing the strategies and support that helped them cope, YouTubers reinforced the idea that mental health problems, even severe ones, can be overcome. They provided a wide range of advice and encouragement to promote mental well-being, such as exercising, relaxing, listening to music, getting enough sleep, practicing self-care, and cultivating self-compassion, while discouraging negative thoughts and self-blame. These pep talks were often preceded by personal stories, where the YouTubers explained how self-reflection on their behaviors, emotions, and goals helped them change their attitudes and develop self-compassion. This implied that the advice was based on personal experience rather than professional expertise. Such ‘means-supporting’ and encouraging messages reflect elements of positive psychology, such as fostering positive emotions, resources, self-esteem, optimism, and resilience (Barry 2009). Our findings align with those of Lind and Wickström (2024), who found that much of the mental health content produced by four female Swedish YouTubers was informed by positive psychology and fit within the dominant health promotion framework, enabling people to gain more control over their mental health (WHO 2004). However, these ‘one-size-fits-all’ recovery tips can be perceived as empowering but also as dismissive, overly optimistic, and placing too much emphasis on their individual responsibility for self-improvement and control over recovery (Adeane and Stasiak 2024).

The YouTubers normalized seeking help by using two primary communication strategies. First, they stressed the importance of talking to someone and not facing mental health challenges alone, highlighting the role of social and peer support. They also shared contact information for accessible services offered by recognized youth mental health organizations, which provide individual counseling and opportunities for peer support. Furthermore, they emphasized the importance of family and loved ones in their recovery. This approach reinforces that social support, relationships, and the broader social context are essential for overcoming mental health challenges, aligning with the mental health promotion framework (WHO 2004, 2020). Second, they recommended therapy as a solution to mental health struggles, normalizing seeking help through therapy. While therapy is generally beneficial at an individual level, YouTubers rarely addressed the challenges of accessing therapy, such as long waiting times, cost, or the therapy process itself. Overall, their descriptions of mental health problems paid little attention to societal and contextual factors, despite these structural factors being integral to mental health promotion (WHO 2004). Moreover, presenting therapy as a straightforward solution to mental health issues has been critiqued through the lens of therapeutic power (Brunila *et al.* 2021), which can obscure structural and societal problems. By focusing on individual responsibility for self-improvement, the responsibility for addressing structural issues may be shifted away from broader societal efforts and placed on the individual. From a mental health promotion, the advice offered by YouTubers appears to support the strengthening of individual resources and personal responsibility in managing life. At the same time, their advice underscores the importance of social support in enhancing mental well-being and facilitating recovery. Emphasizing a community-level perspective could serve as a counterbalance to the dominant health and mental health discourses that tend to prioritize individual responsibility (cf. Wickström and Lind 2024).

### Strengths and limitations

A key strength of this study lies in its data collection method. To the best of our knowledge, no systematic data collection and analysis of YouTube videos focused on mental health content has been conducted on this scale before. Although time-consuming, the data collection process was thorough, enabling an in-depth examination of various phenomena. If a different method had been used, such as limiting the publication period of the videos or searching for content based on vlog titles, a large portion of the vlogs and their mental health-related contents might have been overlooked.

The selection of YouTubers for analysis was based on nominations from young people facing mental health challenges, ensuring that the chosen popular YouTubers were relevant to the mental health context of this study. However, a limitation of using YouTube as a data collection platform is its inherently unstable nature. Vlogs can disappear, as was the case with one YouTuber’s vlogs in this study, preventing a future multimodal review of her content. In addition, the impact of platform algorithms, which prioritize user history and interests, on influencer visibility, audience preferences, and engagement remains unclear. Despite this, influencers must strategically align their content with platform algorithms and operational logic (Abidin 2018).

YouTube, as a platform, differs from other currently popular short-video-based social media platforms like TikTok and Snapchat. While YouTube now includes short videos through YouTube Shorts, it remains primarily a platform for long-form content. Moreover, the participants in our study were selected based on mentions from young people aged 15 and older. Our findings might differ if younger individuals were included. Research on youth and social media has often neglected to consider age and psychosocial development stages, although these factors could influence the reasons for social media use and the ability to critically evaluate platforms and the reliability of information.

### Further research

Further qualitative research on mental health content created by SMIs is needed, particularly on other popular platforms like Snapchat, TikTok, and Instagram, where content and operational logic differ from YouTube. In addition, qualitative research exploring the perceptions of viewers who seek such content could provide valuable insights into this phenomenon. It would also be beneficial to explore quantitative aspects, such as ratings, views, likes, comments, and the contents of comments, using machine learning methods.

## CONCLUSIONS

Despite not branding themselves as mental health educators, popular YouTubers act as role models by sharing narratives of their mental health struggles and recovery with their large, often young audiences. In doing so, they shape public understanding of mental health and its management. YouTubers provide social support, encourage help-seeking, and normalize the pursuit of self-help and relief. However, the self-disclosures, along with the volume and frequency of posts, should be interpreted within the context of their commercial partnerships and the pressure to maintain consistency in their channel profiles.

YouTubers’ approaches to offering advice illustrate two distinct levels of mental health promotion. While encouraging help-seeking and social support highlights the community and social environment, advice on therapy, self-care, and individual responsibility focuses on the individual level of health promotion.

For practitioners working with young people facing mental health challenges, these findings provide insight into the types of social support young people seek and how mental health information can be tailored to better resonate with them. Compared with professionals, SMIs are often more relatable, perceived as more credible, and easily accessible to young people. This presents an opportunity for health promotion organizations to collaborate with YouTubers in campaigns and interventions aimed at engaging young people, a group often difficult to reach through traditional health promotion methods. Such collaborations could play a crucial role in advancing mental health equity.

## Supplementary Material

daaf074_Supplementary_Data

## Data Availability

The data underlying this article will be shared only on reasonable request to the corresponding author.
