# Supplementary material for: Promoting youth well-being: a qualitative study of Finnish YouTubers’ mental health content
Source: Health Promot Int. 2025 Jun 12;40(3):daaf074. doi: 10.1093/heapro/daaf074 (PMC12158547; doi:10.1093/heapro/daaf074)
Supplement: daaf074_Supplementary_Data [file daaf074_supplementary_data.zip › Supplementary File 2_Categorization.docx]

Supplementary File 2. Categorization of the codes.

| Subcategories (codes) | Upper categories |
| --- | --- |
| Addiction; Anxiety; Mentally difficult; Reasons for depression; Depression; Mental health problems and disorders; Feeling bad; Fears | MENTAL HEALTH DIFFICULTIES |
| ADHD; Anorexia; Autism spectrum disorder; Burnout; Depression; OCD; Panic disorder; Psychotic disorders; Eating disorder | DIAGNOSED MENTAL DISORDERS |
| Recommending professional help; Help, support; Help-seeking; Normalizing help-seeking; Pep talks; Recommending literature that supports mental wellbeing; Mental health services; Guidance, advice; Therapy; Encouragement speech | WAYS OF HELPING |
| Everyday life; Emotions and reasons related to separation; Separation; Relationships; Miscarriage; Fear of losing loved ones; Infertility; A child; Intimate relationship; Love; Pregnancy termination; Pregnancy; Sex; Number of sexual partners; Important things in life; Parenthood; Alone as a choice | FAMILY, CHILDREN, EVERYDAY LIFE |
| Life values, priorities; Life management; Self-compassion; Self-knowledge; Development; Gratitude; Self-esteem; Happiness; Positive things; Love; Coping/recovery; Important things in life | FACTORS CONTRIBUTING TO MENTAL WELLBEING |
| (Sexual) harassment; Endometriosis; Physical illness; Pain; Body image; Corona time; Lack of life management; Desperation; Physical harm, cutting; Harassment; Shame; Identity crisis; Self-severity; Self-hatred; Attempted suicide; Self-destructiveness; Self-esteem problems; Envy; Repentance, bad choices; Masking; Weight loss and gain speech; Weight management; Fears; Intoxicants; Quarreling; Social pressure; Stigma; Social exclusion; Eating disorder; Guilt; Appearance; Loneliness; Over-control; Overperforming | FACTORS THAT BURDEN MENTAL WELLBEING |
| Melancholy; Helplessness; Stressing; A sense of disappointment; Feeling of inadequacy; Mourning; Sorrow; Normalization of emotions; Downplaying emotions; Talking about emotions; A difficult subject to talk about; Difficult feelings | THE RANGE OF EMOTIONS AND EMOTIONAL SPEECH |
| Introversion, shyness; The boundary between public and private; Handling of publicity; Publicity; World of social media; Social situations; Criticism of a YouTuber; Comparison with others; Hate speech | PUBLICITY AND LIVING AS A CELEBRITY |
| Burnout; Capability conditions; Lack of motivation; Feeling of inadequacy; Stress; Sleeplessness; Fatigue, exhaustion | STRESS AND COPING AT WORK |
| Commercial cooperation, anti-bullying; Bullying; Emotions related to bullying; Description of bullying; Reasons for bullying; Solving a bullying problem; Acting in a bullying situation | BULLYING |
| Followers' concerns about a YouTuber's health; Peer support for the YouTuber; Thanking for support; Lack of peer support; Peer support | PEER SUPPORT |
| Commercial cooperation, own products; Commercial cooperation, anti-bullying; Cooperation videos and partners | COMMERCIAL COOPERATION |
| Downplaying own concerns/feelings and reassuring followers; Request for followers; Ask to share own experiences; Building a follower relationship; You-form; Information; Reassuring followers; Convincing the followers all OK; Asking for stories; Influencing; Interaction | BUILDING A FOLLOWER RELATIONSHIP |
| Statement; Multimodality; Mental health; Instructions on how to watch and interpret the video; Personality traits; Explanation, why not publish, why the pause; Content warning; YouTuber's role/tasks/goals; Aspects of a YouTuber's work | VLOG CONTENT AND YOUTUBERS’ WORK |
| Concentration; Mental well-being and the health behaviors that promote it; Reflecting on your own actions; Personal experience and expertise; Downplaying own concerns/feelings and reassuring followers; Questioning one's own abilities; Downplaying the problems; Talking, opening up; Coping strategy; Recovery story; Witnessing violence | EXPERIENCE, EXPERTISE, AND ACTION |
